# Effectiveness of Pelvic Proprioceptive Neuromuscular Facilitation Techniques on Balance and Gait Parameters in Chronic Stroke Patients: A Randomized Clinical Trial

**DOI:** 10.7759/cureus.30630

**Published:** 2022-10-24

**Authors:** Manali A Boob, Rakesh K Kovela

**Affiliations:** 1 Physiotherapy, Ravi Nair Physiotherapy College, Datta Meghe Institute of Medical Sciences, Wardha, IND; 2 Physiotherapy, Nitte Institute of Physiotherapy, Nitte (Deemed to be University), Mangalore, IND

**Keywords:** pelvic inclination, gait velocity, proprioceptive neuromuscular facilitation, berg balance scale, palpation meter

## Abstract

Background

Stroke is the second leading reason for death and the third most common reason for disability. Stroke is a source of possible substantial harm and is often more disabling than lethal. Common stroke defects include stiffness, tiredness, loss of balance on the afflicted side, as well as gait impairment, resulting in an inability to sustain postural alignment. Pelvic proprioceptive neuromuscular facilitation (PNF) is a physical rehabilitation that combines functionally dependent diagonal activity patterns with neuromuscular facilitator strategies to improve motor behaviour, endurance, and muscle activity and control. This protocol was created to describe the experimental study design for evaluating the combined impact of pelvic PNF and task-oriented exercises in chronic stroke patients to improve balance and gait parameters.

Aim and objective

The purpose of our study is to investigate the effectiveness of pelvic PNF as well as task-oriented exercises on balance, gait parameters, and in pelvic asymmetry.

Methods

The participants (n=30) were stroke survivors who fulfilled the inclusion criteria for research and were divided into two groups. The regimen lasted four weeks and took 30 minutes each day. Patients were evaluated at the beginning and end of their treatment. In both groups, pre- and post-intervention outcome measures were recorded and the data was analyzed.

Result

Following four weeks of rehabilitation, subjects showed remarkable improvement in balance, gait parameters, and pelvic inclination in both groups, i.e., pelvic PNF and task-oriented exercises in group A and task-oriented exercises in group B, but Group A showed a major improvement in outcome measures. A p-value of less than 0.05 was considered significant. Despite the fact that both treatment regimens were successful for the patient, pelvic PNF combined with task-oriented exercises exhibits a statistically significant difference from task-oriented exercises.

Conclusion

Pelvic PNF along with task-oriented exercises proved to be beneficial and can help in the restoration of balance and gait parameters as a result of normalisation in the geometry and symmetry of the pelvis in stroke patients. The pelvis, which is a connecting link between the trunk and lower limbs, plays a crucial role in balance and also in lower limb performance exclusively in gait.

## Introduction

Stroke is defined as "rapid growing clinical signs of localized disruption of brain activity, with complaints persisting 24 hours or more or fatal consequences, with no evident explanation other than the vascular origin" [1]. In India, stroke is the most widely recognized reason for mortality and disability; the estimated updated overall prevalence of stroke in impoverished areas is 84-262/100,000, while in metropolitan centres it is 334-424/100,000 [2]. According to recent demographic estimates, the prevalence rate is 119-145/per 100,000 people [2]. The type of stroke has a substantial impact on patient survival; hemorrhagic strokes account for the majority of deaths, with mortality rates ranging from 37% to 3% at one month, while ischemic strokes have a mortality rate of just 14.7% at one month [3].

Common stroke defects include stiffness, tiredness, and loss of balance on the afflicted side, as well as gait impairment resulting in an inability to sustain postural alignment [4]. The pelvic region is recognized as a vital critical location during static and dynamic postural shifts, allowing the body to retain momentum and modify weight variations [5]. Brunnstrom discovered, after studying a significant number of hemiplegic patients, that an almost stereotyped series of events occur during rehabilitation following a cerebrovascular injury [6]. A motor function deficit is indicated by hemiplegia on the contralateral side of the injury [7]. Spasticity is a motor condition marked by a velocity-dependent increase in muscle tone and increased stretch resistance; the greater and faster the stretch, the stronger the spastic muscle's resistance [6]. Spasticity is often associated with other neurologic impairments, especially paresis, which makes assessing its effects and treatment outcomes more difficult [6].

There are several techniques and interventions suggested for stroke patients such as proprioceptive neuromuscular facilitation (PNF), strength training programs, task-oriented training, training with visual feedback, a sensory-motor training program, balance training, robotic-assisted locomotor training, locomotor training, Intervention to manage spasticity like PNF, application of a cold pack, massage, electrical stimulation, etc. [3-8]

PNF stands for proprioceptive neuromuscular facilitation, any of the sensory receptors that offer information about the body's movement and location are referred to be proprioceptive, the term "neuromuscular" describes both nerves and muscles are involved, facilitation is the method of developing smoother patterns [9]. PNF is a physical rehabilitation technique that combines functionally dependent diagonal activity patterns with neuromuscular facilitator strategies to improve motor behaviour, endurance, and muscle activity and control [10]. The discovery of this approach was made possible by Kabat, Knott, and Voss' revolutionary work in the 1940s and 1950s [10]. Interpersonal feedback is used in conjunction with some PNF procedures, such as joint approximations, traction, irradiation, and overflow, to enhance muscle activation and motor performance [11].

Task-oriented training is the method of carrying out essential practical exercises or tasks to develop well-organized and efficient motor skills [12]. Task-oriented programming is focused on current motor learning models and the processes paradigm of motor control [12].

## Materials and methods

After receiving consent from the institutional ethics committee of the Datta Meghe Institute of Medical Sciences, deemed to be a University (approved number: DMIMS(DU)/IEC/2021/371) the research was conducted in the neuro-physiotherapy Outpatient Department (OPD), Sawangi, Wardha. The participants were chosen from the neuro-physiotherapy departments of the Acharya Vinoba Bhave Rural Hospital in Sawangi, Meghe, Wardha, Maharashtra, as well as the Ravi Nair Physiotherapy College.

Patients who met the inclusion and exclusion criteria and underwent the necessary evaluation were chosen for this research. The inclusion criteria were patients who experience a period of recovery at least six months after the initial stroke events, patients between 40 to 75 years of age, both males and females, patients who can sit independently, patients with stages 4-5 on Brunnstrom grading, patients with pelvic asymmetry, and patients capable of following commands. The exclusion criteria were patients suffering from severe arthritis, patients with any cognitive dysfunction, patients who have undergone any type of spine or lower limb operation in the span of the last one month, any other neurological abnormalities that impair sensory function, any other cardiopulmonary deficits, patients having fixed deformities or contractures in the spine and lower limb, patients with any type of spine or lower limb fractures or dislocations, patients who are already in a trial. The material required for the study was a printed copy of the data collection sheet, informed consent, a printout of the Berg Balance Scale (BBS), palpation meter, plinth, pillow, obstacles, wobble/balance board, staircase, measuring tape, stopwatch, and chalk. Patients signed written informed consent forms after the objectives and the study approaches were explained to the patients.

**Figure 1:**
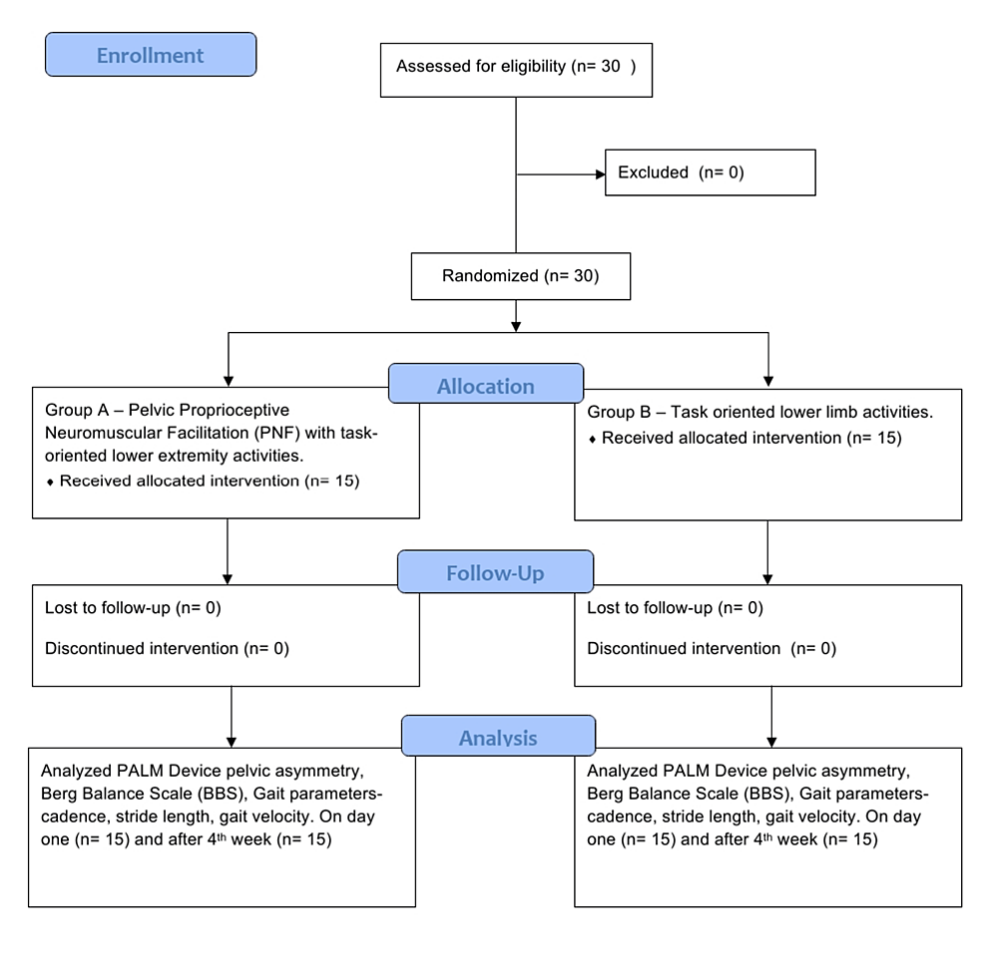
Flow diagram of the study procedure

The participants diagnosed with chronic stroke were randomized through simple random sampling and allocated through the sequentially numbered, opaque, sealed envelope (SNOSE) technique into Group A and Group B. Group A was given pelvic PNF along with task-oriented exercises for lower extremities and Group B was given only task-oriented exercises for the lower limb. Outcome measures were assessed before the beginning of the study and after the completion of the study. Pre- and post-outcome measures were taken by using a palpation meter, BBS, and gait parameters by the assessor who was blinded and aware of the outcome measures. Treatment was planned for 30 minutes each day for six days per week for four weeks. The enrollment, allocation, follow up and analysis are represented in Figure 1. 

Outcome measures

Palpation Meter (PALM) Device 

**Figure 2:**
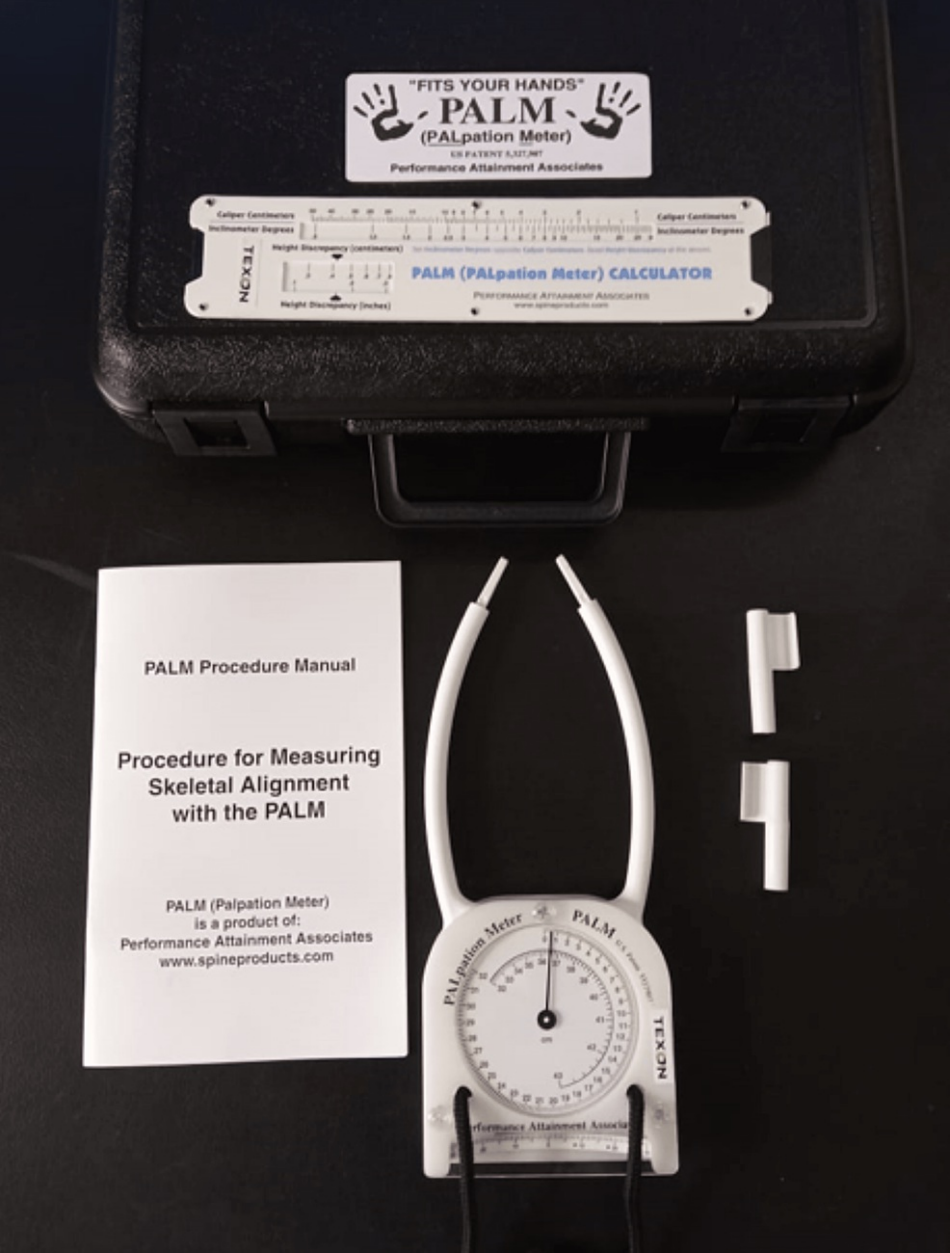
Palpation meter (PALM) device

**Figure 3:**
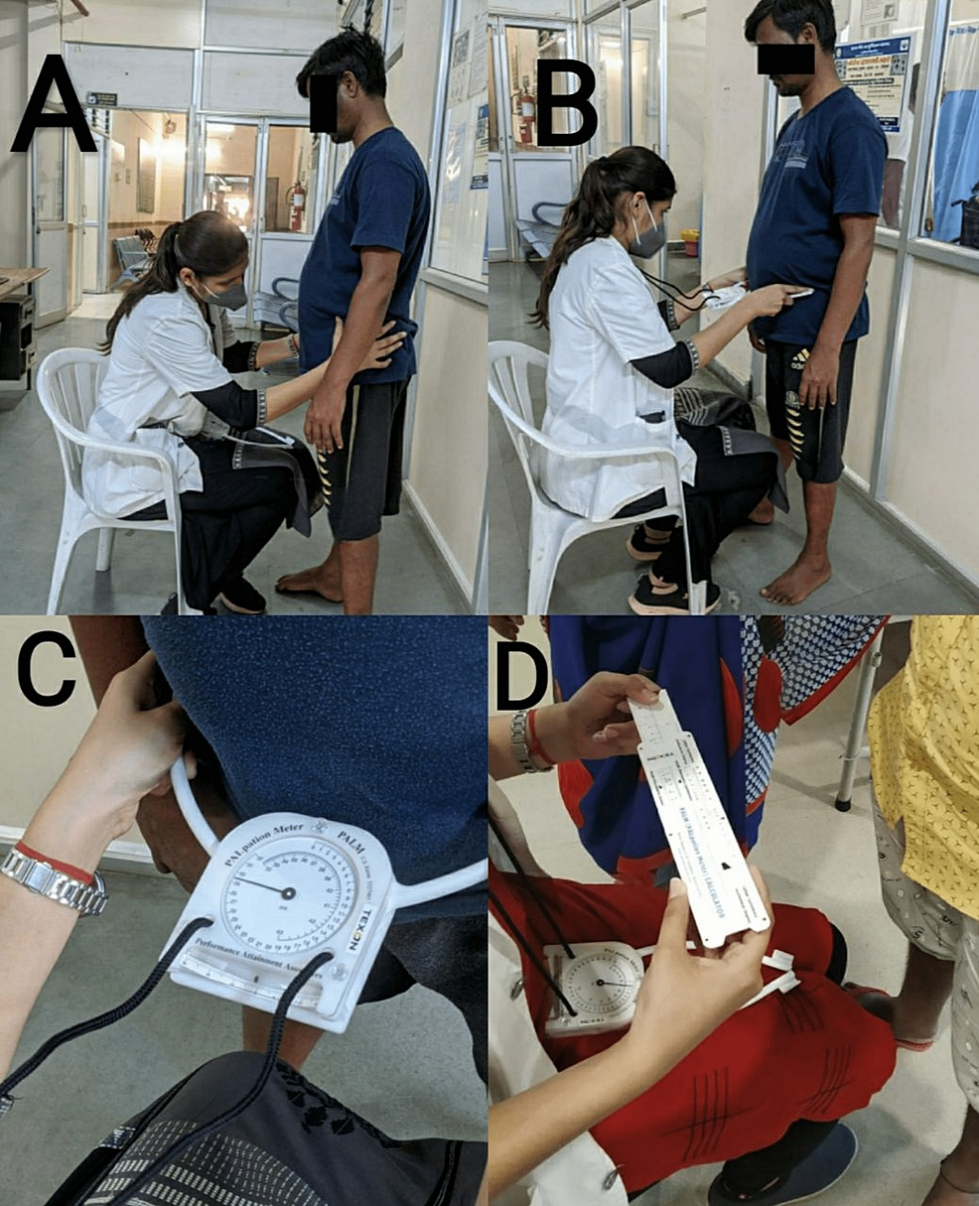
A demonstration of using a palpation meter to check for pelvic asymmetry (A) palpation of the patients' ASIS; (B) measurement of pelvic inclination with palpation meter; (C) calliper dial; (D) palpation meter calculator

The Palpation Meter allows an examiner to test for skeletal asymmetry. The PALM combines the objectivity and reliability of calliper and inclinometer readings with the simplicity and proprioceptive benefits of palpation. Between the two palpating fingers, the calliper measures the distance in millimetres. The inclinometer measures the angle between two palpating fingers in degrees. The height difference between the two landmarks may be measured in centimetres and inches using a special slide rule calculator. The picture of the palpation meter is shown in Figure 2. A demonstration of the palpation meter is shown in Figure 3.

Berg Balance Scale (BBS)

The BBS is a popular clinical scale for assessing a person's dynamic and static state of equilibrium. For practical balancing tests, BBS is commonly known as the gold standard. The test lasts approximately 15-20 minutes and consists of 14 fundamental balance exercises. The ultimate result is the summation of all scores.

Gait Parameters

Stride length: The distance between two successive heel strikes of a similar foot is known as stride length, which is made up of two-step lengths. Cadence refers to the number of steps done in a certain period. Gait velocity by 10-metre walk test is a performance indicator that measures walking speed in m/s over a small distance. Intervention

Interventions

Intervention for Group A

Rhythmic initiation, slow reversal (dynamic reversal), and stabilizing reversal were given for 15 minutes, six days a week for four weeks, coupled with task-oriented lower extremity activities for 15 minutes. Each technique lasted for five minutes with enough rest time offered for each person based on their comfort level. These treatments were performed on the afflicted side, with the hips flexed to 100° and the knees flexed to 45°, these treatments were performed to aid with an anterior pelvic elevation and posterior pelvic depression [5]. A cushion supported their neck. The physiotherapist positioned themself behind the patients, following the plane of the patient’s pelvic movements. For anterior elevation, the therapist's hands were placed just on the subject's anterior iliac spine, and for posterior depression, they were placed on the patient's ischial tuberosity. "Pull up" was employed to help with pelvic anterior elevation, and "push down" and "sit into my hands" helped with pelvic posterior depression [5]. Rhythmic initiation was commenced after the patient voluntarily relaxed, after which the therapist would move the patient passively, later proceeding with assisted movement, then active movement, and finally active-resisted movement. Slow reversal involves a dynamic concentric contraction of strong agonists immediately followed by a dynamic concentric contraction of weak antagonists. Stabilizing reversal is a technique that involves alternate isotonic contractions with enough resistance to keep the body from moving. The therapist permits just a very slight movement in response to the dynamic command. The treatment included PNF features, including placement, manual touch, stretching, resistance, and vocal instructions.

Intervention for Group B

**Figure 4:**
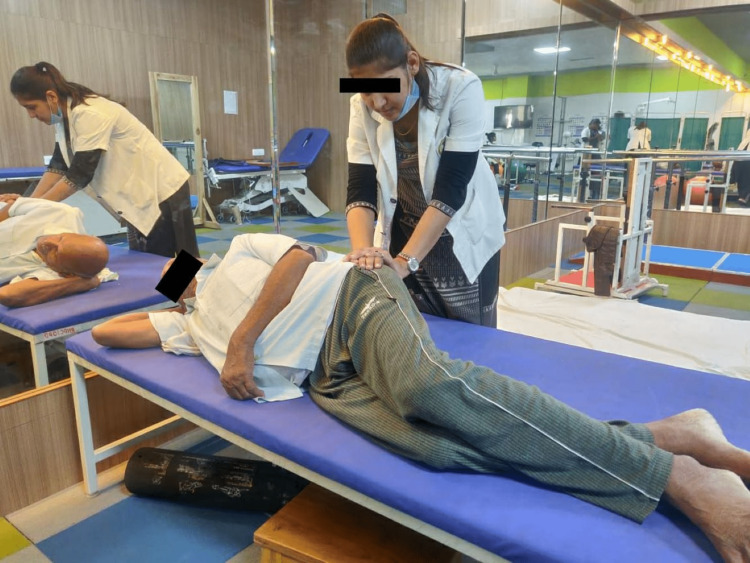
Pelvic PNF demonstrated in the patient PNF: proprioceptive neuromuscular facilitation

**Figure 5:**
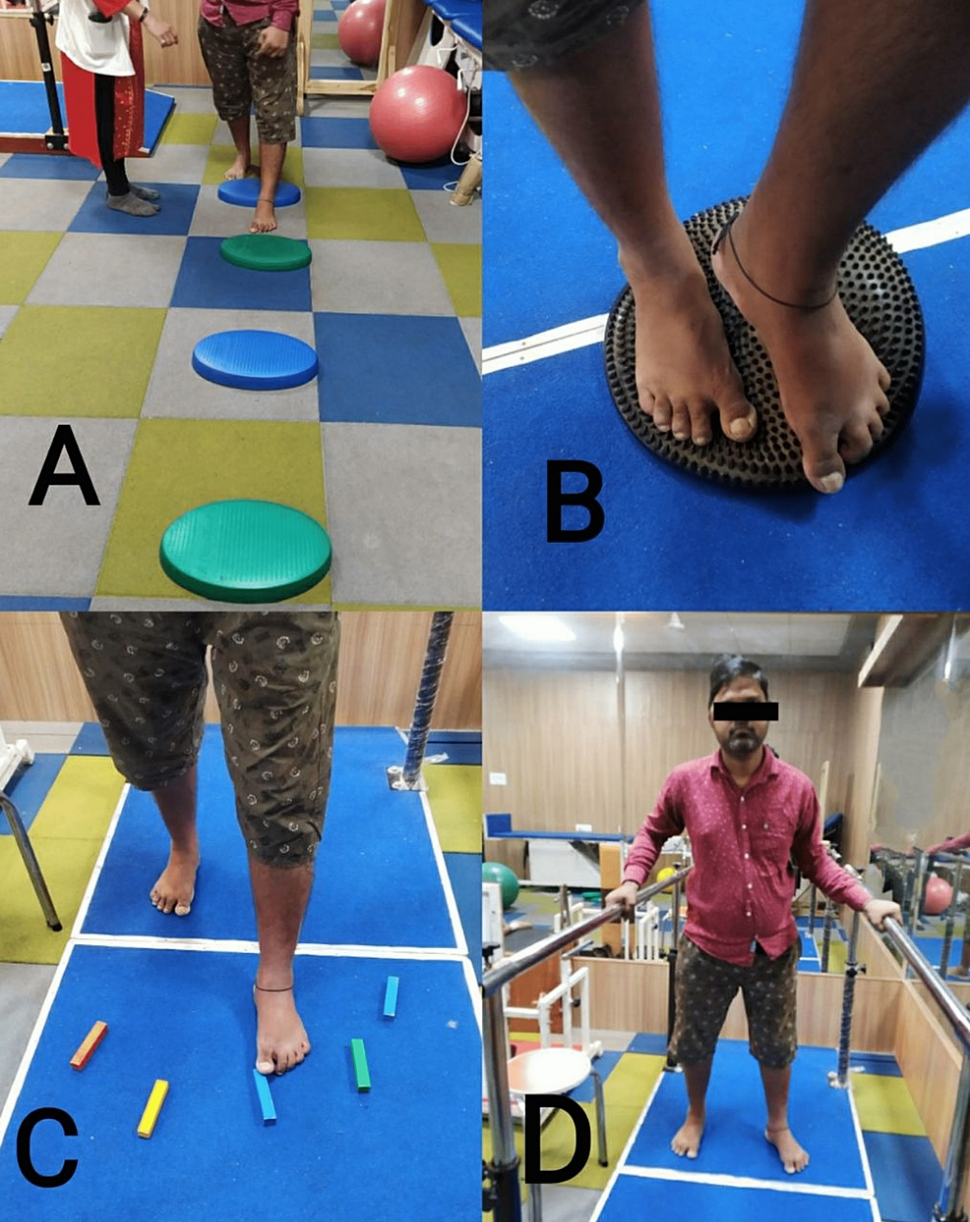
Shows task-oriented exercises. A) obstacle crossing; (B) standing on a wobble board; (C) dragging a foot and placing it on objects; (D) forward walking and backward walking on the parallel bar.

For four weeks, Group B was provided with just task-oriented lower-limb activities for 30 minutes each day, six days per week. Activities for group B include reaching toward an object across the table while standing with symmetrical weight distribution over both legs, standing on a wobble board, forward walking, backward walking, obstacle crossing, walking on an uneven surface, stair climbing, and walking on a ramp. Pre- and post-outcome measures were taken by using PALM, BBS, and gait parameters by the assessor who was aware of the outcome measures. The intervention of groups A and B are shown in Figures 4-5.

Statistical analysis

Data collected were analyzed using descriptive statistical analysis, including a chi-square test, the student's paired and unpaired t-test, and software from the Statistical Package for Social Science (SPSS) 27.0 version (IBM Corp., Armonk, NY) and GraphPad Prism 7.0 version (GraphPad Software, San Diego, CA) with p-value less than 0.05 declared the threshold of effectiveness. The frequency percentages were utilised to characterise qualitative data, while the mean and standard deviation would be used to illustrate quantitative data. The paired t-test was used to compare the effects of pelvic PNF and task-oriented against task-oriented approaches on balance and gait characteristics in patients with continuing stroke. If the data did not follow a normal distribution, the Wilcoxon sign rank test was used.

## Results

**Figure 6:**
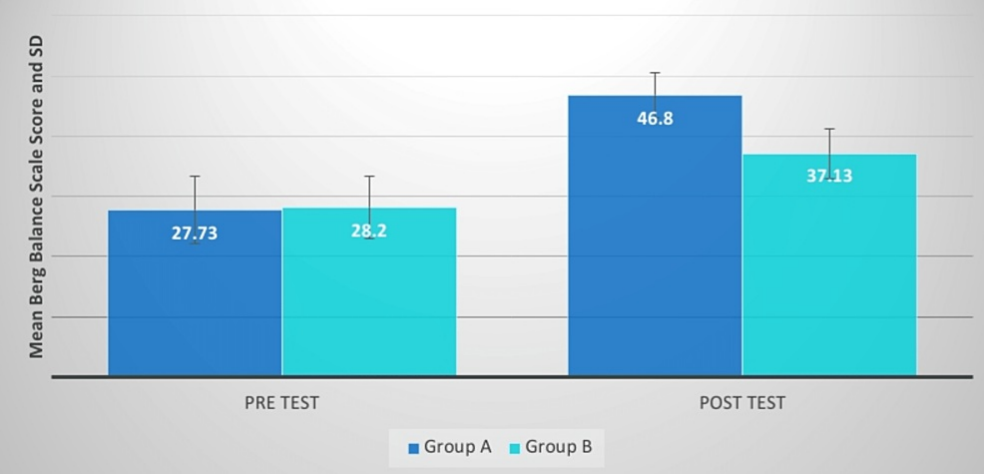
BBS score comparison between two groups before and after treatment BBS: Berg Balance Scale, SD: standard deviation

**Figure 7:**
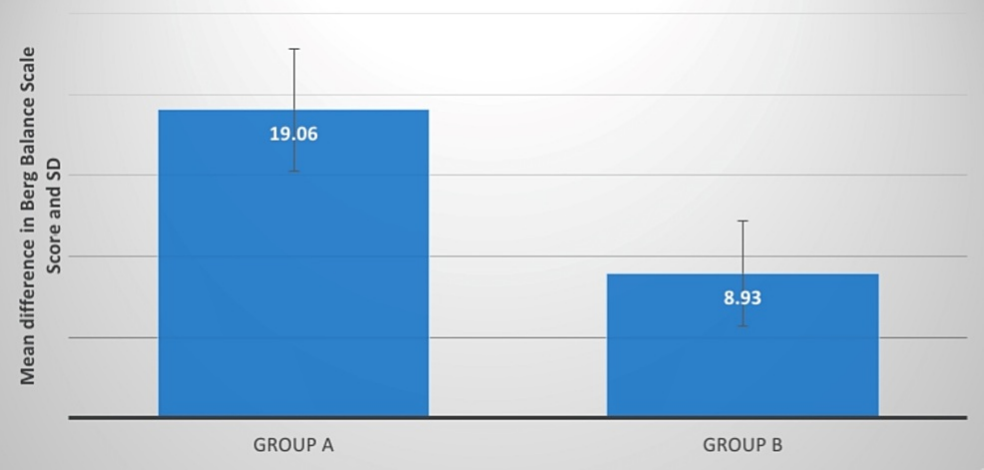
Evaluation of the mean difference between BBS score in two groups BBS: Berg Balance Scale, SD: standard deviation

**Figure 8:**
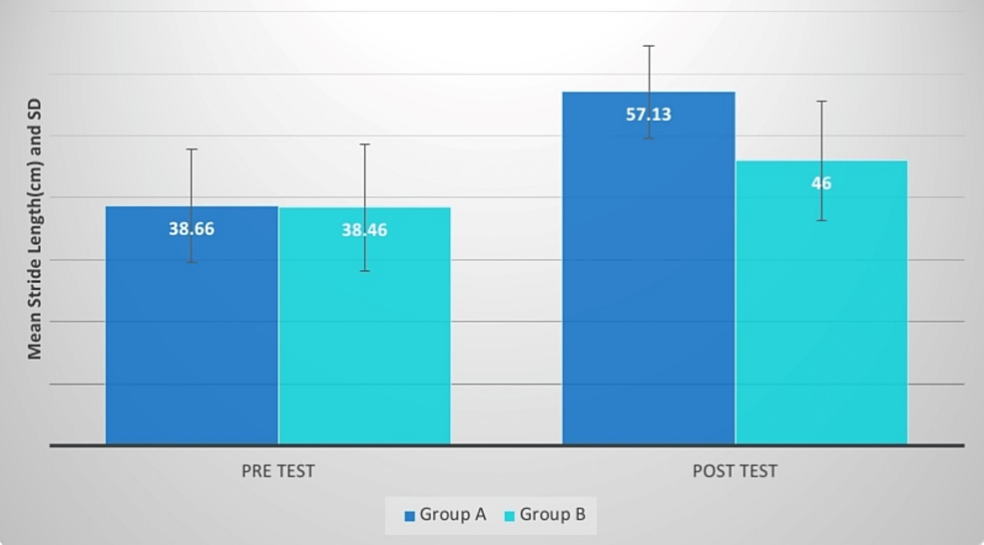
Stride length (cm) comparison between two groups before and after treatment SD: standard deviation

**Figure 9:**
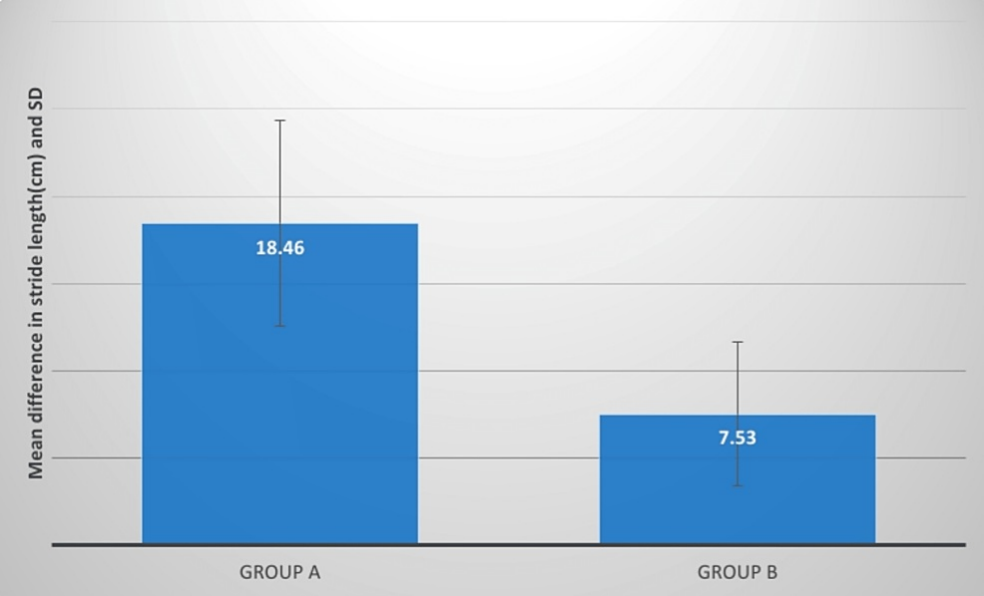
Evaluation of the mean difference between stride length (cm) in two groups SD: standard deviation

**Figure 10:**
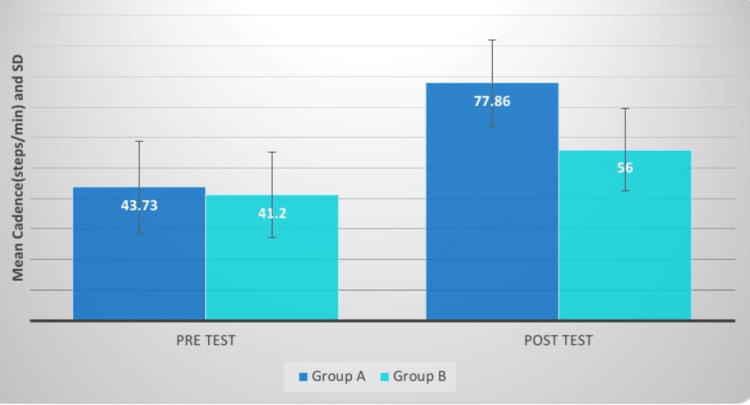
Cadence (m/s) comparison between two groups before and after treatment SD: standard deviation

**Figure 11:**
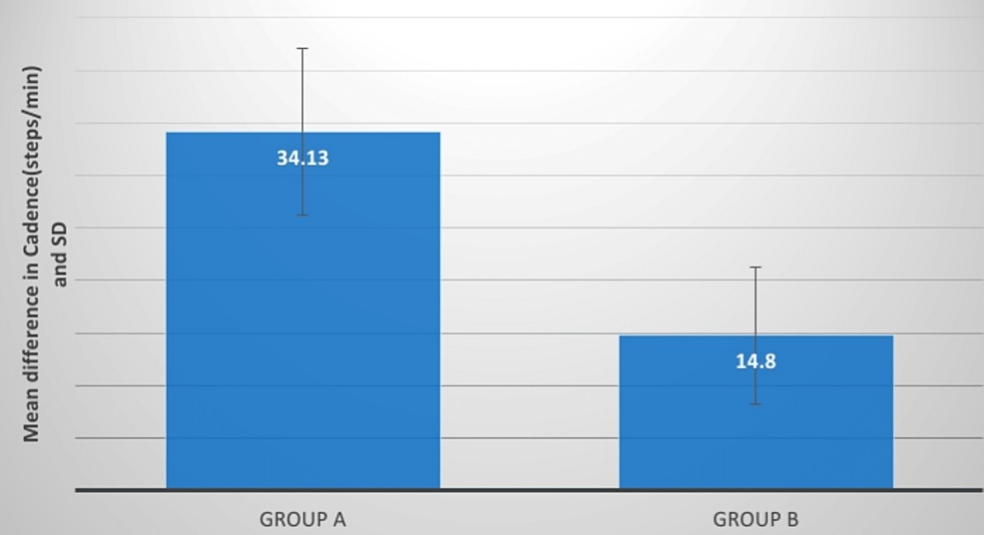
Evaluation of the mean difference between cadence (m/s) in two groups SD: standard deviation

**Figure 12:**
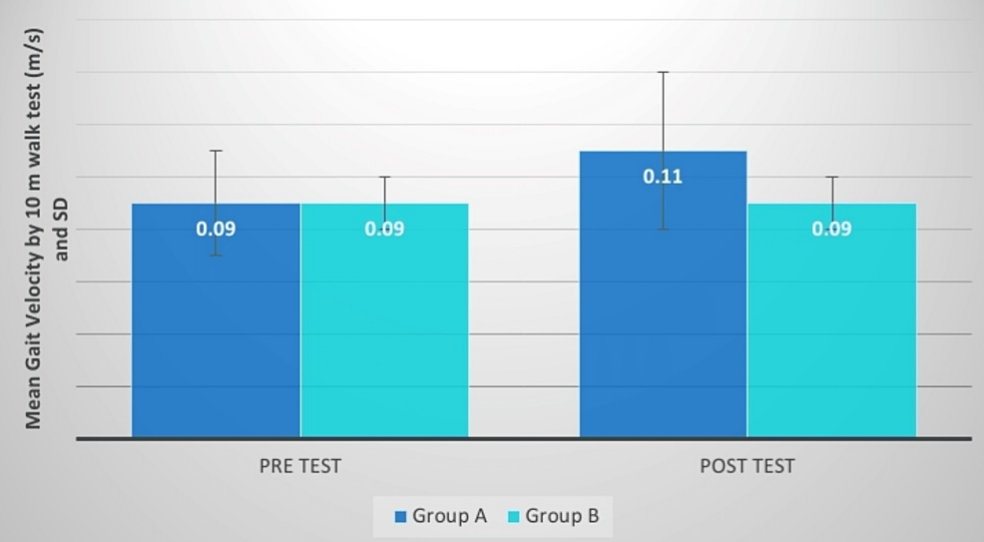
Gait velocity (m/s) comparison between two groups before and after treatment SD: standard deviation

**Figure 13:**
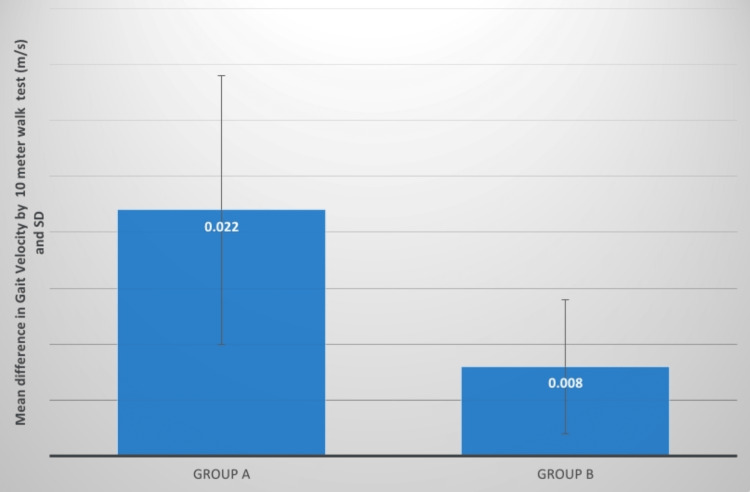
Evaluation of the mean difference between gait velocity (m/s) in two groups SD: standard deviation

**Figure 14:**
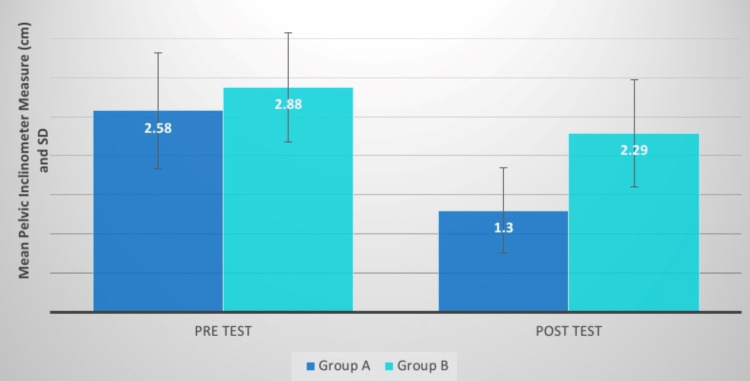
Pelvic inclinometer measure (cm) comparison between two groups before and after treatment SD: standard deviation

**Figure 15:**
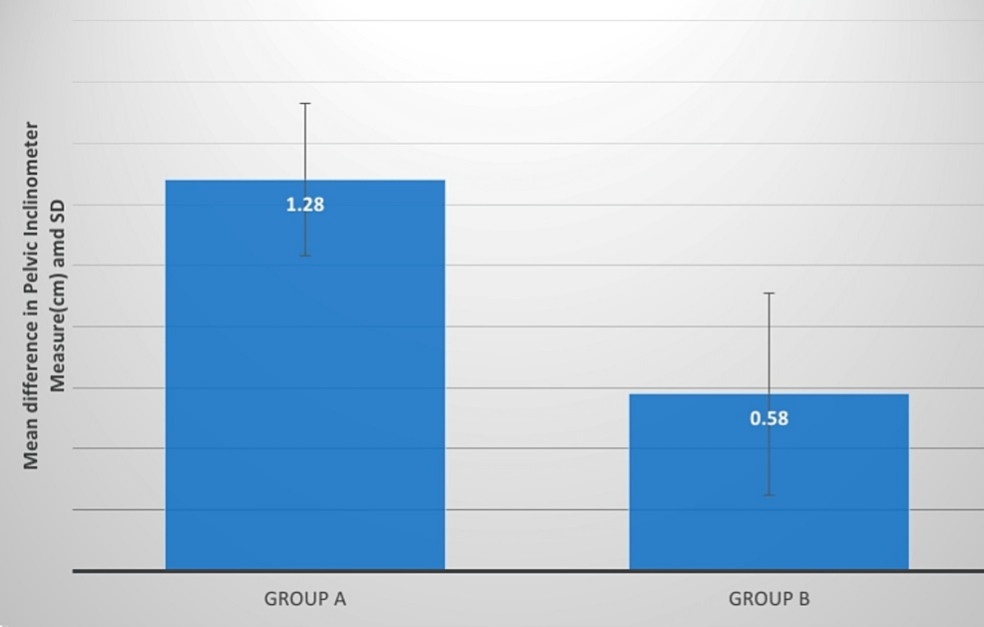
Evaluation of the mean difference between pelvic inclinometer measure(cm) in two groups SD: standard deviation

The effects of the intervention on various outcome measures, including the Berg Balance Scale, stride length, cadence, gait velocity, and pelvic inclination measure, are shown in Table 1 and Figures 6-15, respectively. Table 1 highlights the importance of comparing and post-intervention results for groups. along with the statistical analysis and a mean difference of the variables that were pre-measured. The findings indicate that both groups are statistically significant, but that group A's post-treatment measures are more significant than those of group B. This revealed that group A's protocol would be more appropriate.

**Table 1:** Significance of comparing pre- and post-intervention results for groups s: significant

Outcome Measure	Group A			Group B			Mean difference		
	Pre-treatment	Post-treatment	t-value	Pre-treatment	Post-treatment	t-value	Group A	Group B	t-value
BBS	27.73±5.59	46.80±3.78	19.49; P=0.0001, S	28.20±5.21	37.13±4.15	10.82; P=0.0001,S	19.06±3.78	8.93±3.19	7.91; P=0.0001,S
Stride length	38.66±9.11	57.13±7.43	12.14; P=0.0001,S	38.46±10.21	46±9.62	7.11; P=0.0001,S	18.46±5.89	18.46±5.89	5.89; P=0.0001,S
Cadence	43.73±15.12	77.86±14.05	16.65; P=0.0001,S	41.20±14.10	56±13.40	8.78; P=0.0001,S	34.13±7.93	14.80±6.92	7.28; P=0.0001,S
Gait velocity	0.09±0.02	0.11±0.03	7.69; P=0.0001,S	0.09±0.01	0.09±0.01	6.82; P=0.0001,S	0.022±0.012	0.008±0.004	3.92; P=0.001,S
Pelvic inclination	2.58±0.74	1.30±0.55	19.50; P=0.0001,S	2.88±0.70	2.29±0.69	6.72; P=0.0001,S	1.28±0.25	0.58±0.33	6.35; P=0.0001,S

## Discussion

Transient ischemic strokes were once suggested to be brief, focal neurologic impairments of vascular origin that lasted or less than 24 hours. If a cerebral impairment persisted for longer than seven days, it was considered a stroke. The phrase "reversible ischemic neurological impairment" was used to describe neurological abnormalities that persisted for between 24 hours and seven days when it was proven that the majority of incidents lasting for this time period were associated with cerebral infarction and should be classified as stroke. The previously used phrase was removed from the clinical vocabulary. As a result, the views of stroke in North America and the World Health Organization diverged, with one emphasizing indications of infarction and the other clinical symptoms [13,14]. PNF exercises are particularly efficient in producing voluntary contraction and enhancing functional activities in stroke patients, according to research by Chaturvedi et al [15]. Kumar et al. (2012) PNF's effect on stride length and functional capacities in the hemiplegic. According to studies, hemiplegic patients' gait characteristics and functional skills are significantly impacted by pelvic PNF [16].

The study involves 15 participants in groups A and B. The study's findings illustrate the effect of pelvic PNF and task-oriented lower extremity exercises for chronic stroke patients. The group that received pelvic PNF together with task-oriented lower-limb exercises improved much more than the group that received simply task-oriented lower-extremity activities. Pelvic PNF combined with task-oriented lower extremity exercises was assigned to group A, while task-oriented lower extremity activities were assigned to group B. Five outcomes were utilized in both groups to determine if there was a meaningful recovery or improvement in the patient's condition. On the beginning and final days of the protocol, the outcome measure was evaluated. The Berg Balance Scale and gait variables such as stride length, cadence, gait velocity, and the PALM device for evaluating pelvis misalignment were all utilized to determine the results. The Berg Balance Scale result was computed using a 14-point scale, stride length in cm, cadence in the number of steps per minute, gait velocity in m/s, and pelvic inclination in centimetres.

The Berg Balance Scale (BBS) was administered to the participants in groups A and B. This is a clinical scale for evaluating static and dynamic balance. Group A had a t-value of 19.49, which was higher than group B's t-value of 10.82. Both groups exhibited substantial improvement, while group A demonstrated more impact than group B. The p-value interpretation was significant. The average t-value difference between the two groups was 7.91. Kim et al. examined the effect of aquatic coordinated movement using the PNF pattern on the balance and gait variable where 20 stroke survivors were randomly assigned to one of two groups: experimental or control. Neurodevelopmental therapy was given to both groups of patients, whereas the experimental group used the PNF to conduct synchronized movements underwater. When compared to the control group, the experimental group significantly improved on the BBS, Functional Reach Test, Ten-Meter Walk Test, and Timed Up and Go Test [17].

The stride length of group A patients improved their performance more than group B patients after a four-week treatment. Group A had a higher t-value than group B. The t-value mean difference was 5.89 and the p-value was significant. Patni conducted research to examine the effectiveness of pelvis PNF and hip extensor strength activities on locomotor variables in hemiplegics, with Group A receiving pelvic PNF training and Group B receiving hip extensor strength training. The findings of this study showed that PNF approaches had a substantial impact on gait metrics in hemiplegic individuals [7]. PNF is a way of treating neuromuscular impairment by enhancing the movement of resources, particularly through stimulating proprioceptors [7-18].

The cadence was assessed pre and post-treatment and which was significant in both groups but the outcome of group A is more efficient. Khanal et al. investigated the impact of the pelvic PNF approach on trunk movement stimulation in 30 individuals with hemiplegia who were allocated into two groups randomly. The experimental group underwent pelvic PNF, whereas the control group received truncal exercises. Both groups also got therapy in the form of tonal control and range of motion activities for the afflicted limbs. The intervention was delivered daily for at least four weeks, five days a week. Following therapy, there was an enhancement in outcome variables such as trunk performance, and trunk lateral flexion range of motion as measured by an inclinometer, balance, and gait metrics [19].

To begin calculating gait velocity, a 10 m distance is measured in the hallway. The patient is then asked to walk at his or her normal pace without falling, and the timeframe is recorded in seconds. The velocity was then calculated, and the distance covered was calculated as 10 m divided by the time it took to complete the distance. Readings were recorded on Day 1, prior to the start of therapy, and they were nearly identical in both groups. Post-intervention both the groups are significant but the group A value is greater than B. The mean difference in t-values is 3.92, and the mean difference in p-values was significant. Dubey et al. analyzed the effects of hip muscle strengthening, gait speed, and activities of daily living after stroke on the trunk and lower extremity mobility function. The experimental class received pelvic stability exercises whereas the control class received normal therapy for 60 minutes three to four times a week for approximately six weeks. Pelvic stabilization training has been believed to enhance hip muscular strength, gait velocity, and control of the trunk and lower extremities in stroke patients [20].

Comparing groups, A and B before and after therapeutic interventions, the mean difference in pelvic inclination between them was 1.28 0.25 and 0.58 0.33. The p-value was 0.0001 and the mean t-value difference was 6.35. There was a remarkable decrease in the value of pelvic inclination in group A. Petrone et al. conducted research to evaluate the Palpation Meter's accuracy and dependability. This study included 15 healthy subjects and 15 afflicted subjects with suspected lower limb discrepancy (LLD). The clinician used the PALM to measure the pelvic height difference. A simply standing anteroposterior radiograph of each patient's pelvis was collected, and the pelvic difference from the radiograph was measured to compare with PALM values. ICCs (intraclass correlation coefficients) were computed. The results of PALM compared with radiographs show that the evaluating pelvic tilting and inclination discrepancies, the PALM is a dependable, valid, and accurate tool [21].

The research was limited in that it was only done on a small population. This shows that a larger population needs to participate in the same study to see whether PNF combined with task-oriented activities is useful for stroke patients. There was no prolonged monitoring of patients, and the study only lasted a few weeks. In future research, the number of therapy sessions should be increased for a quicker recovery.

## Conclusions

The main objective of this research is to investigate the combined therapeutic efficacy of pelvic PNF techniques as well as task-oriented approaches to balance and gait parameters, as well as in the treatment of pelvic asymmetry in a stroke survivor. The study's outcome measures are the PALM device, BBS, and gait parameters. The PALM device was used to check pelvic asymmetry, which was the most reliable instrument used in the study rather than the traditional radiographic measurement. BBS helped to assess the balance and gait parameters; an increase in cadence led to a rise in the individuals' average stride length, resulting in an improved gait pattern. The impactful output of this research shows that the pelvic PNF technique with task-oriented activities of the lower limb in chronic stroke patients was more beneficial than only task-oriented activities. This results in improving their balance, gait parameters, and pelvic asymmetry. The recovery of the patient was faster, which improved their personal quality of life. An outcome of this research was the learnings gleaned from both therapies. The data were examined with a paired t-test and reported as a research article.
